# Risk factors associated with nonalcoholic fatty liver disease evaluated by elastography in patients with type 2 diabetes

**DOI:** 10.20945/2359-3997000000492

**Published:** 2022-06-02

**Authors:** Roselee Pozzan, Ronaldo Gama Pena, Cátia Cristina Silva Sousa Vergara Palma, Raquel de Carvalho Abi-Abib, Carlos Terra, Roberta Arnoldi Cobas

**Affiliations:** 1 Universidade do Estado do Rio de Janeiro Serviço de Diabetes Disciplina de Diabetes Rio de Janeiro RJ Brasil Disciplina de Diabetes/Serviço de Diabetes, Universidade do Estado do Rio de Janeiro, Rio de Janeiro, RJ, Brasil; 2 Universidade do Estado do Rio de Janeiro Unidade de Fígado Divisão de Gastroenterologia Rio de Janeiro RJ Brasil Divisão de Gastroenterologia – Unidade de Fígado, Universidade do Estado do Rio de Janeiro, Rio de Janeiro, RJ, Brasil

**Keywords:** Type 2 diabetes mellitus, nonalcoholic fatty liver disease, controlled attenuation parameter (CAP), elastography

## Abstract

**Objective:**

There is controversy about the indication for nonalcoholic fatty liver disease (NAFLD) screening in patients with type 2 diabetes mellitus (T2D). The present study aims to contribute to NAFLD surveillance in patients with T2D, assessing the association of clinical and biological variables with hepatic stiffness and steatosis.

**Subjects and methods:**

A cross-sectional design was used, with data collection from electronic medical records, including adults with T2D who underwent transient elastography (TE) between June 2018 and December 2019. Liver stiffness and steatosis were evaluated using TE and controlled attenuation parameter (CAP), respectively, with cutoff points > 8 kpa for increased stiffness and > 275 dBm for steatosis. The relationship between clinical variables and elastography results were evaluated by bivariate correlation and multivariate analysis, using SPSS 27. Seventy-nine patients (n = 79) met the inclusion and exclusion criteria.

**Results:**

Advanced fibrosis and hepatic steatosis were detected in 17,7% and in 21,5% of the patients, respectively. There was a direct and significant correlation between CAP and BMI, waist circumference, HbA1c, triglycerides levels, and insulin doses and an inverse correlation with HDL. The waist circumference, low levels of HDL cholesterol and the insulin dose maintained a significant association with CAP values in multivariate analysis. Elastography values showed an inverse correlation with HDL and a direct correlation with BMI and insulin dose. The association was only maintained for the insulin dose in multivariate analysis.

**Conclusion:**

Our results suggest that clinical factors such as insulin dose, waist circumference, and HDL cholesterol levels could identify T2D patients more likely to present NAFLD.

## INTRODUCTION

Nonalcoholic fatty liver disease (NAFLD) is defined by the fat accumulation in hepatocytes without a secondary cause, such as alcohol consumption, use of steatogenic drugs or genetic syndromes (1). It is a pathological condition with a large clinical spectrum with stages ranging from a simple fatty infiltration, inflammation of the liver parenchyma (nonalcoholic steatohepatitis) to advanced fibrosis, cirrhosis and, in some cases, hepatocellular carcinoma (2,3). Steatosis, without steatohepatitis, does not seem to confer an increased risk of progressing to cirrhosis or hepatocellular carcinoma (4). The prevalence of NAFLD rises in parallel with global increases in obesity and diabetes, and is the most frequent cause of liver disease today (5). In people with type 2 diabetes (T2D), the prevalence of NAFLD varies from 30 to 75% (6,7), and fibrosis prevalence ranges from 15% to 40% depending on the diagnostic method, cutoff points and the population studied (7,8). A significant clinical and economic burden due to NASH with T2DM over the next 20 years are predicted by mathematical models (9).

There is a bidirectional relationship between diabetes and NAFLD. People with T2D and NAFLD have a higher risk of progressing to steatohepatitis, cirrhosis and death. On the other hand, NAFLD seems to be associated with a higher risk of progressing to diabetes and its macro and microvascular complications (5). The presence of steatosis in T2D patients seems to be associated with greater insulin resistance (IR), worse glycemic control and dyslipidemia (7). Diabetes mellitus, central obesity and NAFLD seem to have common pathogenic mechanisms (5), with NAFLD described as the hepatic component of metabolic syndrome (MS) (10). Furthermore, cardiovascular disease is the main cause of death in patients with NAFLD (6,11,12), as observed in T2D patients and patients with metabolic syndrome (12).

The scientific society's guidelines are controversial about NAFLD screening in patients with T2D (4,13) due to uncertainties related to diagnostic means and treatment. In addition, screening for NAFLD in patients with T2D has not proven to be cost-effective, and there is a lack of studies on its long-term benefit (4). Some guidelines proposed the screening of NAFLD in patients with obesity or MS by using ultrasound and biochemical markers (13-15), while others do not recommend systematic screening, although they recognize the increased risk in this group who therefore should be maintained under surveillance (5,14).

There are different methods used to assess NAFLD. Liver biopsy is the “gold standard” for the assessment of steatosis and fibrosis and the only method capable of identifying the presence of steatohepatitis (7). However, it is a high-cost invasive procedure with a risk of complications. Thus, noninvasive methods for quantifying steatosis and fibrosis have been used, such as imaging methods and the use of scores for prediction the presence of steatosis or fibrosis (16). Among the most widely used tests, abdominal ultrasonography (USG) has been used frequently because it is a relatively low cost exam, but it has limited sensitivity to detect steatosis when there is < 20% of liver fat accumulation (17). Transient elastography (TE) is a noninvasive, reproducible, and easy to perform method, developed to assess the degree of hepatic stiffness that correlates with the degree of fibrosis. The controlled attenuation parameter (CAP) is a methodology that allows for an ultrasound wave attenuation assessment during elastography exam. The CAP provides a numerical value that shows a good correlation with the histological degree of steatosis and has been recently used to assess the presence and degree of steatosis in different studies (18).

The present study aims to contribute to NAFLD surveillance in patients with T2D, assessing the association of clinical and biological variables with hepatic stiffness and steatosis.

## SUBJECTS AND METHODS

**Figure 1:**
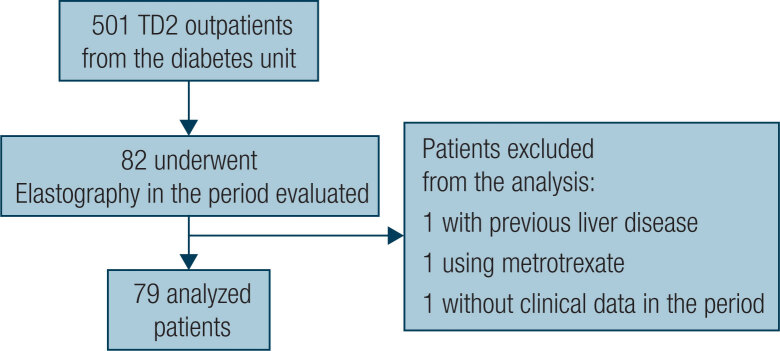
Flowchart of study sample selection.

This is a cross-sectional study with electronic medical data of the Diabetes Unit outpatients from Policlínica Piquet Carneiro, Rio de Janeiro State University (UERJ). The study included patients with T2D who underwent TE between June 2018 and December 2019. The exclusion criteria included previous history of liver disease, history of alcohol abuse or use of steatosis-inducing drugs described in the medical record (Figure 1). The study was approved by the Ethics Committee under CAEE (Certificate of Presentation for Ethical Appreciation) number 00399018.8.00005259.

A single experienced professional performed liver elastography, using Fibroscan^®^ (Echosens, Paris, France). The median value of 10 successful acquisitions, with a success rate of at least 60% and an interquartile range of less than 30% expressed in kilopascals (kpa), was used to represent hepatic stiffness. Cutoff points for the definition of steatosis and advanced fibrosis were CAP > 275 dB/m and hepatic stiffness ≥ 8 kpa, respectively (16).

The following clinical variables were evaluated: sex, age, duration of diabetes, levels of glycated hemoglobin, use of insulin and dose, use of statin and/or fibrate, history of hypertension, systolic and diastolic blood pressure levels, history of cardiovascular disease (CVD, including myocardium infarction or stroke), degree of cardiovascular risk (CVR) (15), waist circumference (cm) and presence of MS, as well as BMI (kg/m^2^) and its classification as normal (18.5 to <25), overweight (25 to <30) or obesity (≥30) (19). According to the International Diabetes Federation (IDF) criteria, increased waist circumference is mandatory for the diagnosis of MS (>90 cm in men and >80 cm in women in South America). As all patients had diabetes, the presence of hypertension or altered levels of HDL or triglycerides were sufficient for the diagnosis of MS (19).

Glomerular filtration rate (GFR) was calculated by CKD-EPI formula (20). The urine albumin-to-creatinine ratio (ACR) was also measured, with patients divided into those with ACR < 30 and ≥30 mg/g and those with ACR < 300 and ≥ 300 mg/g. Screening for diabetes retinopathy (DR) was done by mydriatic binocular indirect ophthalmoscopy (BIO), using the TOPCON^®^, TRC NW 8 ImageNet Lite system, with a single ophthalmologist reading the image. DR was defined as present when at least one eye was affected by any degree of injury. Diabetes neuropathy and protective sensitivity of the feet were evaluated by the distal polyneuropathy assessment scale, as validated by Moreira and cols. (21), and with 10 grams monofilament, according to the methodology described in the Ministry of Health Manual (22), respectively.

Statistical analysis was performed using SPSS version 27 (SPSS, Inc., Chicago, Illinois, USA). Distribution of variables was tested for normality using the Kolmogorov-Smirnov test. An exploratory analysis was performed and continuous variables with normal distribution were expressed as mean and standard deviation, but those with asymmetric distribution were expressed as median (interquartile range). Categorical variables were expressed as frequencies. Student's t-test or Mann-Whitney test was used to compare means or medians between groups, when indicated. When comparing three groups, one-way analysis of variance (F) or Kruskal-Wallis (Z) was used according to the normality of the analyzed variable. In the same way, a bivariate Pearson or Spearman correlation was used to assess the correlations between the studied variables and the CAP and elastography values. Variables with p value < 0.1 were included in the linear regression multivariate analysis, in which variables without normal distribution were converted to logarithmic scale to fit the model. The multicollinearity test between variables included in the multivariate analysis was performed. A two-sided p value < 0.05 was considered statistically significant.

## RESULTS

Eight two patients underwent liver elastography between June 2018 and December 2019. Seventy-nine met the inclusion criteria. Table 1 shows the clinical and epidemiological characteristics of the patients. The frequency of hepatic steatosis and advanced fibrosis were 21,5% and 17,7%, respectively.

**Table 1:** Characteristics of the patients

Variables		n = 79
Age (years)		61.2 ± 8.5
Sex (feminine), n (%)		50 (63.3)
DM duration (years)		16.8 ± 8.1
HbA1c (%)		8 (6-12)
Insulin use, n (%)		58 (73.4)
Insulin dose (IU/kg/day)		0.7 ± 0.4
Statin use, n (%)		74 (93.7)
Fibrate use, n (%)		4 (5.1)
Tabagism, n (%)		7 (8.9)
Hypertension, n (%)		64 (81)
SBP (mmHg)		132 (120-150)
DBP (mmHg)		79 (70-86)
BMI (kg/m²)		29.8 (27.7-34.3)
BMI classification, n (%)		
	Normal	10 (12.7)
	Overweight	32 (40.5)
	Obesity (Class I; II; III)	21 (26.6); 14 (17.7); 2 (2.5)
Waist circumference (cm)		101.7 ± 12.1
Metabolic syndrome, n (%)*		67 (93.1)
LDL (mg/dL)		75(62-98)
HDL (mg/dL)		47 (40-62)
Triglycerides (mg/dL)		134 (91-196)
Peripheral Neuropathy, n (%) **		18 (22.8)
Protective Sensitivity of the feet (absence), n (%) **		12 (16)
GFR (mL/min/m2)		82 (62-100)
GFR < 60 mL/min/m2) n (%)		16 (20.3)
ACR (mg/g)***		15.45 (8.1-46.0)
ACR ≥30 mg/g, n (%)		22 (32.4)
ACR ≥300 mg/g, n (%)		6 (8.8)
Diabetes Retinopathy, n (%) ****		19 (27.1)
History of MI and/or Stroke, n (%)		16 (20.3)
CV risk score, n (%)		
	Low/intermediate	0
	High	63 (79.7)
	Very high	16 (20.3)
Elastography (kpa)		6 (5-7)
CAP values (dB/m)		230.0 ± 52.5

DM: diabetes mellitus; HbA1c: glycated hemoglobin; SBP: systolic blood pressure; DBP: diastolic blood pressure; BMI: body mass index; LDL: low-density lipoprotein; HDL: high-density lipoprotein; GFR: glomerular filtration ratio; ACR: albumin-to-creatinine ratio; MI: myocardial infarction; CV: cardiovascular; CAP: controlled attenuation parameter. Date present as mean ± DP or median (interquartile range).

* Seven patients not classified (absence of hypertension and triglycerides and HDL values within the normal range with the use of statin and/or fibrate)

** Four patients with no data on the assessment of neuropathy or protective sensitivity of the feet described in medical records

*** Eleven patients without data of ACR in medical records

**** Eight patients without retinography results due to technical impossibility of performing the exam.

There were no significant differences in CAP values according to sex; diabetes duration (< 10 or ≥10 years); HbA1c values (<7% or ≥7%); hypertension; neuropathy or altered protective sensitivity of the feet; and subgroups with ACR <30 or ≥30 mg/g or ACR <300 or ≥300 mg/g. Higher mean CAPs were observed in patients using insulin compared to those who did not (237.4 ± 60.4 *vs.* 209.6 ± 50.0, *p* = 0.04, respectively) and in those with MS compared to those without (233.2 ± 51.8 *vs.* 209.6 ± 50.0, *p* = 0.004, respectively). When comparing patients with normal BMI, overweight and obesity, significant differences were observed in CAP means between groups (176.7 ± 37.5 *vs.* 220.7 ± 52.5 *vs.* 252.4 ± 43.2, respectively, *p* < 0.001). Patients with GFR < 60 had lower CAP means compared to patients with GFR ≥60 (205.4 ± 45.6 *vs.* 236.2 ± 53.6 *p* = 0.03, respectively). Individuals of normal weight or overweight had lower values of elastography than those with obesity [5 (3-8) *vs.* 5(3-27) *vs.* 6 (4-35), *p* = 0.012)]. No significant difference was observed when the elastography values were compared between the groups described above.

There was a direct and significant correlation between CAP and BMI, waist circumference, HbA1c and triglycerides levels and insulin dose; and there was an inverse correlation between CAP and HDL. The elastography values showed an inverse correlation with HDL levels and a direct correlation with BMI and insulin dose. Table 2 describes the correlation between diabetes variables and CAP and elastography values.

**Table 2:** Bivariate correlation between diabetes variables and CAP and elastography values

	CAP (steatosis)		Elastography (hepatic stiffness)	
	r	p	r	*p*
Age	-0.17	0.12	0.07	0.49
BMI	0.45	**<0.001**	0.27	**0.01**
WC	0.42	**<0.001**	0.21	0.05
GFR	0.21	0.06	0.06	0.59
ACR	-0.15	0.22	-0.04	0.70
HbA1c	0.29	**0.008**	0.07	0.50
DM duration	-0.08	0.45	0.06	0.56
LDL	0.07	0.53	0.09	0.40
TG	0.27	**0.01**	0.11	0.31
HDL	-0.31	**0.005**	-0.26	**0.01**
SBP	0.02	0.83	0.01	0.90
DBP	-0.05	0.60	-0.07	0.53
Insulin dose	0.37	**0.005**	0.30	**0.02**

BMI: body mass index; WC: waist circumference; GFR: glomerular filtration ratio; ACR: albumin-to-creatinine ratio; HbA1c: glycated hemoglobin; DM: diabetes mellitus; LDL: low-density lipoprotein; TG: triglyceride; HDL: high-density lipoprotein; SBP: systolic blood pressure; DBP: diastolic blood pressure. r = correlation coefficient.

In the multivariate linear regression analysis only waist circumference, insulin dose and HDL levels remained in the CAP model (Table 3), which explained 35% of the findings. In the elastography analysis, only the insulin dose remained in the model, however, explaining just 19% of the findings.

**Table 3:** Multivariate linear regression including diabetes variables and CAP or elastography values

	CAP (steatosis)		Elastography* (hepatic stiffness)	
	r	p	r	p
WC	0.24	**0.04**		
BMI*			0.19	0.14
Insulin dose	0.28	**0.03**	0.28	**0.03**
GRF*	0.15	0.20		
HDL*	-0.30	**0.02**	-0.24	0.06
TG*	0.03	0.84		
HbA1c*	0.01	0.90		

R^2^ = 0.35, p < 0.001 (CAP model). R^2^ = 0.19, p < 0.01 (elastography model). WC: waist circumference; BMI: body mass index; GFR: glomerular filtration ratio; HDL: high-density lipoprotein; TG: triglyceride; HbA1c: glycated hemoglobin. r = partial correlation coefficient in multivariate linear regression.

* Logarithmic variables used in the models.

## DISCUSSION

The present study shows an association between NAFLD and MS variables, notably the waist circumference and low HDL levels. There was also a direct association with the use of high doses of insulin.

In our study, the frequency of hepatic steatosis and advanced fibrosis were 21,5% and 17,7%, respectively. A recently published study (23), with 307 T2D patients using the CAP found a steatosis prevalence of 73.3%, but the cutoff point used was >233 dB/m, below the one used in the present study. In the same study, the presence of fibrosis identified by elastography was 22.5%, superior to that observed in our study but the cutoff point was also lower (>7 kPa). Another study with T2D outpatients in the USA, found a 70% prevalence of steatosis and 14.8% of fibrosis, using similar elastography cutoff points (8). The population of this last study had twice as many patients with obesity and about half of patients using insulin compared to our study (8).

As for the relationship between obesity, MS and NAFLD, our findings confirm what is described in the literature (7,23,24). CAP and elastography values were higher in individuals with obesity and CAP values were directly related to waist circumference values and inversely related to the HDL. Tuong and cols. (23) evaluated NAFLD using elastography and CAP and found a higher prevalence of steatosis in the greater BMI range. In addition, patients with steatosis had a higher prevalence of hypertension, obesity, central obesity and MS, as well as higher blood glucose and triglycerides and lower HDL. Cruz and cols. (24) described an association between the degree of steatosis and the IR measured by insulin levels and HOMA IR. In another study with T2D patients, the presence of NAFLD was associated with more severe hyperinsulinemia and higher IR in muscle, adipose tissue and liver (25). Recently, the use of the term metabolic dysfunction associated fat liver disease (MAFLD instead of NAFLD) was suggested, considering that the first terminology more accurately reflects the disease pathogenesis (26).

In the present study, CAP and elastography values were directly related to the insulin dose. Since patients who need higher doses of insulin are likely to have more IR, this result is in agreement with other studies that have described the association between NAFLD and more severe IR in T2D patients (27,28). Obesity and fat accumulation in the liver and extrahepatic tissues are considered important determinants of IR, which explain the need for higher doses of insulin and worse glycemic control (7). However, in our study, the correlation between CAP and HbA1c levels disappeared after adjustment. Obese and overweight patients usually need higher insulin doses for glycemic control, however high insulin doses aggravate obesity, creating a vicious circle (29).

NAFLD has been associated with an increased risk of microvascular complications and CVD in T2D patients, but published data are heterogeneous. Ciardullo and cols. (30) found that biomarkers of steatosis were associated with a higher prevalence of albuminuria while those of fibrosis with a higher prevalence of GFR < 60 mL/min/m^2^ and CVD. With the use of elastography and CAP, Yeung and cols. found that albuminuria risk (values above the cutoff point) was higher in patients with advanced fibrosis (31 and Lombard and cols. found a higher risk of CVD and microvascular complications in patients with hepatic fibrosis (32). In our study, we did not find an association between NAFLD and microvascular complications or CVR. The negative relationship, close to the statistical significance found between the CAP and the GFR, disappeared after adjustment in the multivariate analysis. Considering the objectives of this work and unlike the studies cited above, the analysis was not performed comparing T2D patients with or without hepatic impairment, but rather inversely evaluating the values of CAP and elastography in T2D patients with or without micro or macro vascular complications. Furthermore, it is possible that our negative findings are related to the size and characteristics of the studied population.

The present study has several limitations. The associations obtained are at most moderate, the sample size is small and the exclusion of other causes of liver disease was based only on patient's medical records. The patients of the present study were from a specialized outpatient clinic at a tertiary care center, with prolonged disease duration, high prevalence of microvascular complications and high cardiovascular risk. Therefore our findings may not apply to populations with different characteristics. No causal relationship can be ascribed to the associations found, which requires prospective studies with larger samples. On the other hand, the associations found between clinical characteristics easily identifiable in medical practice and the presence of steatosis and/or fibrosis can help to determine which patients with T2D benefit the most from NAFLD diagnostic tests.

Our study contributes to the study of NAFLD in people with T2D with the use of CAP and elastography, considering that those methods have been increasingly used in literature. As controversies still exist about the screening of NAFLD in T2D patients, our results suggest that clinical factors such as insulin dose, waist circumference, and HDL cholesterol levels could identify T2D patients more likely to present NAFLD. However, studies with a larger number of patients are needed to confirm these results and possibly identify the cutoff points for each of the variables that would indicate screening for NAFLD.
